# *De novo* talin-1 variant L353F connects multifaceted clinical symptoms to alterations in talin-1 function

**DOI:** 10.1042/BCJ20253128

**Published:** 2025-09-17

**Authors:** Muktesh Athale, Neil Ball, Latifeh Azizi, Irene Valenzuela, Marta Codina, Andrea Martin-Nalda, Vasyl V. Mykuliak, Rolle Rahikainen, Benjamin T. Goult, Paula Turkki, Vesa P. Hytönen

**Affiliations:** 1 Faculty of Medicine and Health Technology, Tampere University, Tampere, 33520, Finland; 2 Department of Biochemistry, Cell & Systems Biology, Institute of Systems, Molecular & Integrative Biology, University of Liverpool, Liverpool, L69 7ZB, U.K; 3 Clinical and Molecular Genetics Area, Vall d'Hebron Hospital, Medicine Genetics Group, Vall d'Hebron Research Institute (VHIR), Barcelona, Spain; 4 Pediatric Infectious Diseases and Immunodeficiencies Unit, Children's Hospital, Vall d'Hebron Barcelona Hospital Campus, Barcelona, Spain; 5 Fimlab Laboratories, Tampere, 33520, Finland

**Keywords:** focal adhesion kinase, missense mutation, paxillin, skin disease, TLN1, wound healing

## Abstract

Talin-1 is a central integrin adapter protein connecting cytoplasmic domains of integrins to the cytoskeleton. These talin-1-mediated mechanical linkages are crucial for cellular functions such as cell movement and connections with other cells. Here, we report a patient carrying a missense variant, L353F, in the talin-1 head which is associated with a complex set of symptoms, including skin lesions, blood cell abnormalities, and congenital cataracts. We conducted structural and cellular characterization of this variant. Recombinant talin-1 F2F3 fragment with the corresponding mutation showed a decrease in thermal stability and decreased solubility. Reconstitution of talin-deficient cells with L353F talin-1 revealed decreased cell migration velocity, defects in wound healing capacity, and changes in recruitment of the focal adhesion complex protein paxillin. We also observed decreased levels of activated integrin in cells expressing the talin-1 variant, while integrin-binding affinity was preserved as determined biochemically. These observations suggest that changes in integrin adhesion complex dynamics reflect cellular processes and the multifaceted patient phenotype.

## Introduction

Central cellular functions such as cell movement, fate, and differentiation are all dependent on the cell–extracellular matrix (ECM) communication enabled by specialized cellular substructures called focal adhesions. Focal adhesions mediate the connection between the cell exterior and interior and function as machineries for both mechanical and chemical signaling. Through integrin adhesion complexes, cells can sense biophysical characteristics of the extracellular space, such as stiffness, composition, and elasticity. Extracellular mechanical cues such as pressure and shear are also largely transmitted via focal adhesions, enabling cells to adapt and differentiate. The importance of alterations in this cell–ECM communication in the onset and progression of different diseases has been discussed in detail in recent reviews [1–3].

Talin-1, a central component of the integrin adhesion complex, is an adaptor protein involved in the cytoskeletal coupling and activation of integrin adhesion receptors. Talin-1 is composed of an atypical FERM domain, head region, and an elongated rod domain composed of 13 α-helical domains, R1-R13 [4]. Reversible unfolding of the talin-1 rod domains under mechanical force regulates the domain interactome, thus transducing mechanical cues into biochemical signals. Talin-1 has been heavily studied, and we have a reasonably good understanding of its structure [4,5], main binding partners [6], and its capacity to respond to mechanical load by altering its interactions with other molecules such as vinculin [7,8]. However, despite the essential role of talin in cell-ECM communication, its disease associations are only just emerging.

While the association of talin with cardiovascular diseases has gained most attention, recent studies suggest that altered talin functions can cause a much broader array of symptoms than previously thought, and talin has been linked with several diseases [9,10] summarized here. i) Changes in talin-1 and talin-2 expression levels have been associated with the onset and poor prognosis of several cancers, such as prostate cancer and breast cancer [11,12]. ii) A cancer-associated talin-1 isoform has been identified, causing alteration of the mechanostability of talin-1 [13]. iii) There is emerging evidence for the association of talin with the maintenance of endothelial barrier function [14] and a talin-1 splice-site variant identified from patients with familial systemic capillary leak syndrome destabilizes endothelial barrier permeability [15]. iv) Talin-1 induces smooth vascular muscle cell proliferation and migration, and its expression has been found to be down-regulated in the media of aortic dissection samples [16]. Further analysis revealed that talin-1 variants associated with spontaneous coronary artery dissection cause significant changes in essential cellular functions, such as migration and adhesion assembly [17]. v) Talin down-regulation increases the risk of cardiomyocyte hypertrophy [18] and vi) atherosclerotic plaques [19] in coronary artery disease. vii) High plasma levels of soluble talin-1 are associated with the severity of coronary artery disease [20]. viii) Talin-1 is essential for the slow rolling and arrest of neutrophils along the endothelium during inflammation [21].

Recently, we identified a patient with a *de novo* talin-1 variant, P229S, that affects talin-1-integrin signaling and is associated with complex clinical symptoms [22]. Here, we report another patient with a novel talin-1 variant, L353F, that is localized close to the integrin-binding site in F3. The complex medical history of this patient coupled with mild but clearly measurable effects on protein structure and function further reinforces the idea that striking medical symptoms can arise from subtle perturbations in talin-1 function. These findings further lay the foundations for the detection of disease-associated talin-1 variants that have been omitted previously.

## Results

### Identification of *de novo* talin-1 variant L353F

The proband is the second child of healthy, non-consanguineous parents. An older sibling has no relevant health issues. During pregnancy, increased nuchal translucency was detected in the first trimester, and an invasive test was performed, revealing a normal karyotype (46, XY). At 21 weeks, right hydrothorax was detected, and a prenatal genetic study using microarray was also completed, which showed normal results. A thoraco-amniotic shunt was placed. In the follow-up ultrasound, migration of the shunt and recurrence of the pleural effusion were observed. Therefore, at 24 weeks of gestation, a second shunt was placed. The shunt was again lost, and the pleural fluid accumulation reappeared. As a result, at 33 weeks of gestation, a therapeutic thoracocentesis was performed. Due to the fetal pathology, labor was induced at 38 weeks of gestation. The delivery was vaginal, with an Apgar score of 9/10. The somatometry at birth was as follows: weight 2910 g (−0.59 SD), length 48.5 cm (−0.58 SD), and occipitofrontal circumference (OFC) 33.5 cm (−0.29 SD). The infant was admitted to the intensive care unit for observation but did not present any respiratory complications. A follow-up blood test revealed leukopenia and thrombocytopenia, with a minimum of 4 × 10⁹/L leukocytes and 61.5 × 10⁹/L platelets. Serial monitoring showed progressive improvement, with final counts at discharge of 4.6 × 10⁹/L leukocytes, 1.3 × 10⁹/L neutrophils, and 111.6 × 10⁹/L platelets.

During the first years of life, he experienced multiple episodes of acute bronchitis, mostly managed on an outpatient basis with bronchodilators and corticosteroids, without presenting other respiratory complications. Leukopenia persisted, with counts ranging between 1.96 × 10⁹/L and 4.92 × 10⁹/L leukocytes, neutrophils between 1.3 × 10⁹/L and 3.8 × 10⁹/L, and lymphocytes between 0.5 × 10⁹/L and 1.0 × 10⁹/L.

Regarding the thrombocytopenia detected at the neonatal level, since it persisted, a bone marrow aspiration was performed at six months of age, which did not reveal any significant abnormalities. It was considered peripheral thrombocytopenia (with a negative autoimmunity workup) of unknown etiology. At the age of ten, a bone marrow study was repeated, indicating normal cellularity with no signs of dysplasia, suggesting a peripheral pathology. In the analytical controls performed to date, platelet counts have ranged between 64 × 10⁹/L and 112 × 10⁹/L, with a normal mean platelet volume.

At the age of nine, during an ophthalmological examination, a congenital cataract was detected in the left eye. Psychometric development has progressed without issues. The child is currently 13 years and 4 months old, with no problems in schooling or neurobehavioral disorders.

**Figure 1:**
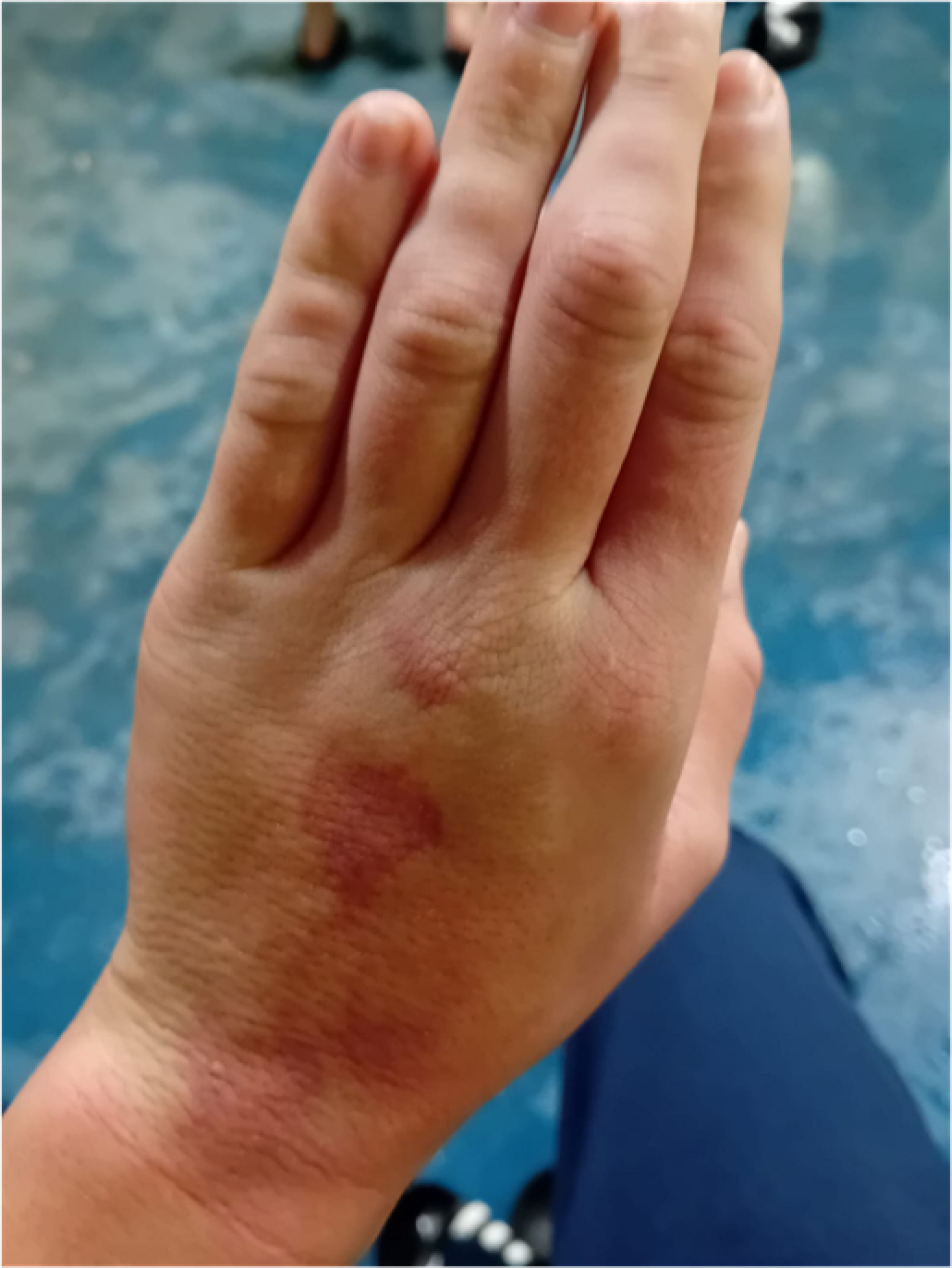
Talin-1 L353F is associated with complex symptoms, including skin lesions and skin swelling. Swollen hands with skin lesions were observed after being exposed to cold weather. Full list of symptoms is provided in Table 1.

Recently, skin lesions and swelling in the dorsum of the hands have been observed when the child has played football in cold weather (Figure 1). The skin spots disappear in less than an hour, but the swelling persists for two to three days. While the swollen hands cannot be moved well, there is no pain associated with these symptoms.

**Table 1:** Clinical manifestations associated with talin-1 mutations. Medical conditions observed in patients with talin-1 variants P229S [22] and L353F (this study). Human phenotype ontology (HPO) terms are included for clinical manifestations where available

Clinical manifestations associated with P229S	Clinical manifestations associated with L353F
**Skin related** Eczematoid dermatitis (HP:0000964) Localized skin lesion (HP:0011355) (Hyperpigmented lesions) Recurrent cutaneous fungal infections (HP:0011370) Keloid-like skin changes (HP:0001064)	**Skin related** Eczematoid dermatitis (HP:0000964) Edema of the dorsum of hands (HP:0007514)
**Immune system related** Thrombocytopenia (HP:0001873) Leukopenia (HP:0001882) Immunodeficiency (HP:0002721) IgG deficiency (HP:0004315) T lymphocytopenia (HP:0005403) Atypical common variable immunodeficiency	**Immune system related** Thrombocytopenia (HP:0001873) Leukopenia (HP:0001882)
**Other** Congenital cataract (HP:0000519) Recurrent bronchitis (HP:0002837) Arthritis (HP:0001369) Arthralgia (HP:0002829) Hematuria (HP:0000790) Migraine (HP:0002076) Intestinal lymphangiectasia (HP:0002593) Tick-like body jerking Hearing impairment (HP:0000365) Elevated circulating hepatic transaminase concentration (HP:0002910) Recurrent sinusitis (HP:0000437) Recurrent otitis media (HP:0000403)	**Other** Congenital cataract (HP:0000519) Recurrent bronchitis (HP:0002837)

Besides the prenatal tests conducted, exome sequencing (ES) was performed, which identified a heterozygous variant in the *TLN1* gene. The variant c.1057C > T, p.(L353F) causes a change from leucine to phenylalanine at position 353 of the protein. The segregation study in peripheral blood samples from the patient’s parents indicated that it is a *de novo* variant. The L353F change has not been described in the general population database gnomAD v4.1, nor has it been reported in the literature. The *in silico* metapredictor REVEL [23], which integrates conservation and protein structure parameters, gives it a score of 0.71, suggesting that it could be deleterious to its function. AlphaMissense [24] predicts the variant to be likely pathogenic (0.977) and PolyPhen-2 [25] predicted the variant to be probably damaging (0.999).

Informed consent was obtained from the parents to perform genetic analyses and complementary studies, as well as to publish the clinical and molecular data and also to share pictures.

### Mutated talin-1 F2F3 shows reduced thermal stability

**Figure 2:**
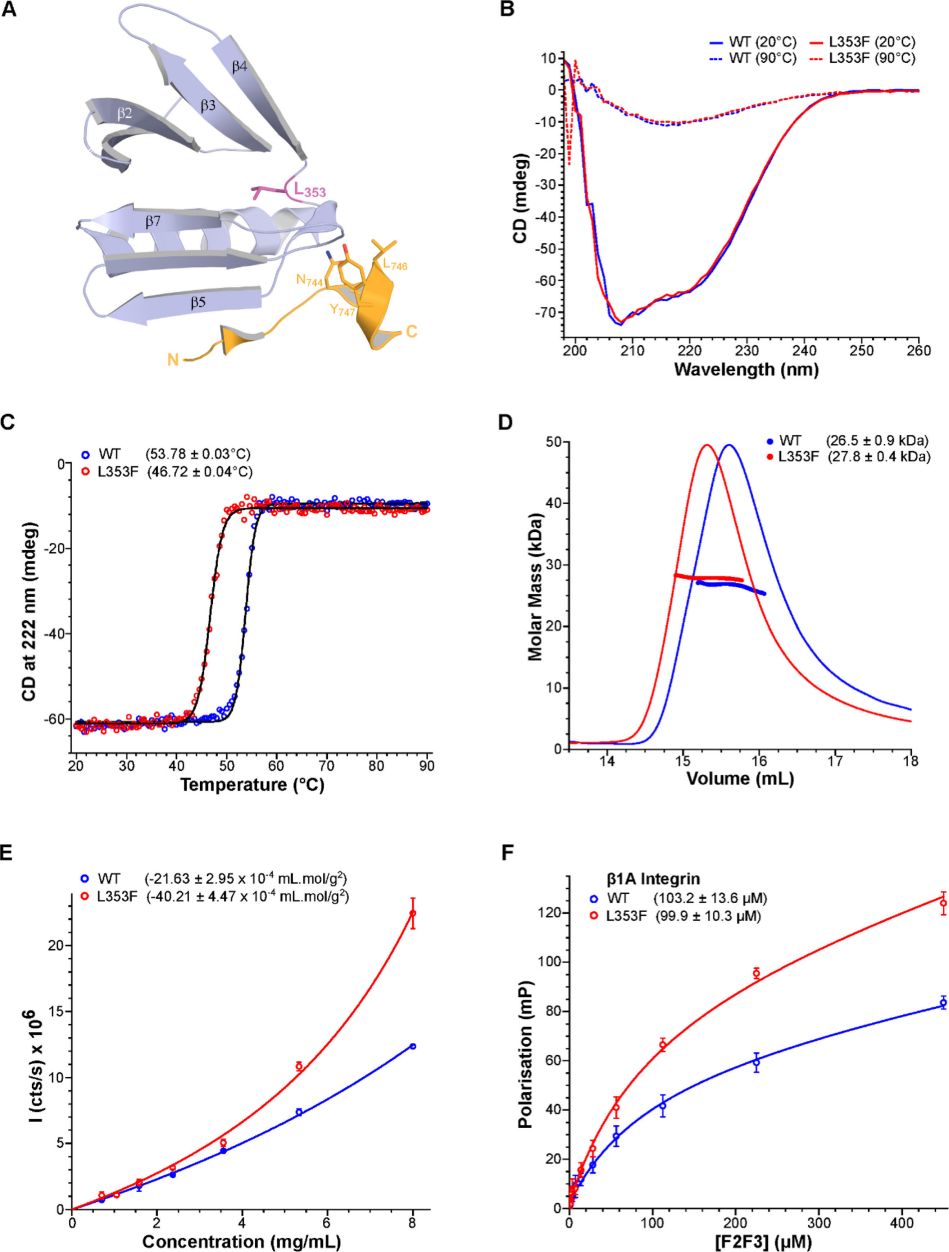
The L353F mutation in F3 of talin-1 reduces the stability of the F2F3 double domain. **(A**) L353 (purple) is in the linker between the two β-sheets (β1–4 and β5–7) in F3 (blue) and is proximal to the binding site that recognizes the NPxY motif in β-integrin cytoplasmic tails (orange). Structure modeled in PyMOL using the β3 integrin-talin-1 F2F3 chimera structure (1MK7 [26]). (**B**) Circular dichroism spectra of WT (blue) and L353F (red) F2F3 prior to (solid line) and after (dashed line) thermal denaturation. Prior to thermal denaturation, both proteins exhibit very similar spectra indicating that the two are both folded in solution. (**C**) Thermal denaturation of WT (blue) and L353F (red) F2F3. The L353F mutant protein has a melting temperature ~ 7°C below the WT protein, indicating that this point mutation is sufficient to significantly destabilize the double domain module. Melting temperatures shown in parentheses. (**D**) SEC-MALS of WT (blue) and L353F (red) F2F3. Protein at 4 mg/ml was injected onto an S200 10/300 increase gel filtration column. The molar mass of the species present in each peak is shown in parentheses. (**E**) A 1.5-fold dilution series of WT (blue) and L353F (red) F2F3 protein was analysed by static light scattering (SLS) using a Prometheus Panta (NanoTemper). The 2^nd^ virial coefficient (**B_22_**) value (shown in parentheses) was determined, which shows that the L353F protein is more aggregation prone than WT. (**F**) Fluorescence polarization assay measuring the binding of the β1A integrin cytoplasmic tail to talin-1 F2F3 revealed that the mutation only minimally perturbs integrin binding. WT shown in blue and L353F in red. *K_d_* values are shown in parentheses.

The mutated residue is located in the F3 subdomain in the talin-1 head very close to the integrin-binding site (Figure 2A). The side chain of residue 353 is mostly buried in the hydrophobic core of F3 and interacts with the C-terminal helix that connects to the mechanosensitive rod domains via an unstructured linker [27,28]. Talin-1 head subdomains F2 and F3 share a large protein-protein interface (440 Å^2^), and they have been found to have structural co-operativity [26,29] behaving as a single double domain module. The integrin binding site is located within the F3 subdomain, and the F2 subdomain contains a cluster of positive residues that form the membrane orientation patch [29]. Synergy between the F2 membrane binding and F3 integrin binding is critical for robust and co-ordinated integrin activation [29,30]. We and others have previously used the F2F3 subdomains to assess the consequences of mutations, and our previous study covering talin-1 P229S also exploited this strategy [22]. Expression and purification of wildtype (WT) and L353F variant versions of the F2F3 double domain fragment enabled us to compare the biophysical and biochemical properties. Both the WT and L353F versions of the F2F3 protein expressed well and were ≥95% pure following affinity and cation exchange chromatography. ^1^H,^15^N TROSY nuclear magnetic resonance (NMR) spectra of the talin-1 F2F3 WT and L353F proteins confirmed that both proteins are similarly folded (Supplementary Figure S2A) as the peaks were well dispersed and showed considerable similarities between the two proteins. However, as expected, the point mutant resulted in some peaks in F3 being shifted as a result of changes in the local environment around the larger phenylalanine side chain in the L353F variant (Supplementary Figure S2B). Circular dichroism (CD) spectroscopy also indicated no differences in secondary structure (Figure 2B). However, the L353F variant reduced the thermal stability of F2F3 by ~7°C (WT: T_m_ = 53.75°C; L353F: T_m_ = 46.72°C; Figure 2C).

This decrease in thermal stability of L353F, without seemingly affecting either the fold or the secondary structure content, led us to investigate whether the mutation altered the propensity of F2F3 to self-associate. Size exclusion chromatography with multi-angle light scattering (SEC-MALS) was used to determine the molar masses of the WT and L353F F2F3 proteins. The calculated masses agreed with the theoretical molar mass of F2F3 (WT: 26.5 kDa, L353F: 27.8 kDa, theoretical: 28.2 kDa; Figure 2D). However, the L353F eluted slightly earlier than the WT (Figure 2D), suggesting a subtle change in hydrodynamic radius. For an extended view of SEC-MALS data, please consult Supplementary Figure S3. To test whether there was any concentration-dependent self-association/aggregation of the two proteins, we used static light scattering (SLS). We measured SLS across a range of protein concentrations and determined the second virial coefficient (B_22_), which is a measure of protein self-association in solution (Figure 2E). L353F exhibited a more negative B_22_ value than the WT protein (WT: −21.63 × 10^−4^ ml**·**mol/g^2^; L353F: −40.21 × 10^−4^ ml**·**mol/g^2^), indicating that the mutant has a higher propensity for self-association/aggregation. Finally, we used dynamic light scattering (DLS) analysis across a range of concentrations (Supplementary Figure S4) which confirmed a concentration-dependent increase in hydrodynamic radius for the L353F variant, supporting the conclusion that the mutant is slightly less well behaved and more aggregation-prone or weakly self-associating.

As the L353F mutation is in the F3 domain close to the integrin-binding site, we next wanted to test whether the mutation impacted integrin binding. We used a fluorescence polarization (FP) assay to quantify the binding affinity for a β1 integrin peptide. Here, the fluorescein-labeled peptide is titrated with increasing amounts of talin-1 F2F3 protein, and the change in FP as a function of concentration allows the binding constant (*K_d_*) to be determined. Comparison of WT and L353F showed no significant difference in the F2F3 binding affinity for the β1 integrin peptide (Figure 2F). Consistent with the concentration-dependent self-association of the L353F F2F3 seen by SEC-MALS (Figure 2D) and SLS (Figure 2E), we saw increased polarization at higher concentrations compared with WT, suggesting increased self-association.

### All-atom molecular dynamics simulations predict minor effects of the L353F mutation on F2F3 and its affinity for integrin β3

In our previous studies, we developed a pipeline to integrate computational analysis of the effects of variants on protein stability and interactions into our variant characterization analysis. So, we next conducted all-atom molecular dynamics (MD) simulations to estimate the changes in thermodynamic stability upon L353F substitution. We calculated the free energy changes of the mutant F2F3 double domain compared with the WT for F2F3 alone and in complex with integrin β3. This computational analysis predicted a slight decrease in the stability of F2F3 upon introduction of the L353F mutation (by 5.4 ± 1.2 kJ/mol), which is in line with the observed decrease in thermal stability of the F2F3 from the CD data (Figure 2C). MD analysis suggested that the mutation preserves the binding affinity of F2F3 for integrin β3 (mild increase in affinity by -3.2 ± 1.5 kJ/mol was observed), which is also in agreement with the FP assay (Figure 2F). This broad agreement between the experimental and computational data on the effects of the variant further supports the use of MD simulations within a pipeline for evaluating new talin-1 variants.

### Talin-1 L353F mutation influences cell movement but not cell morphology

**Figure 3:**
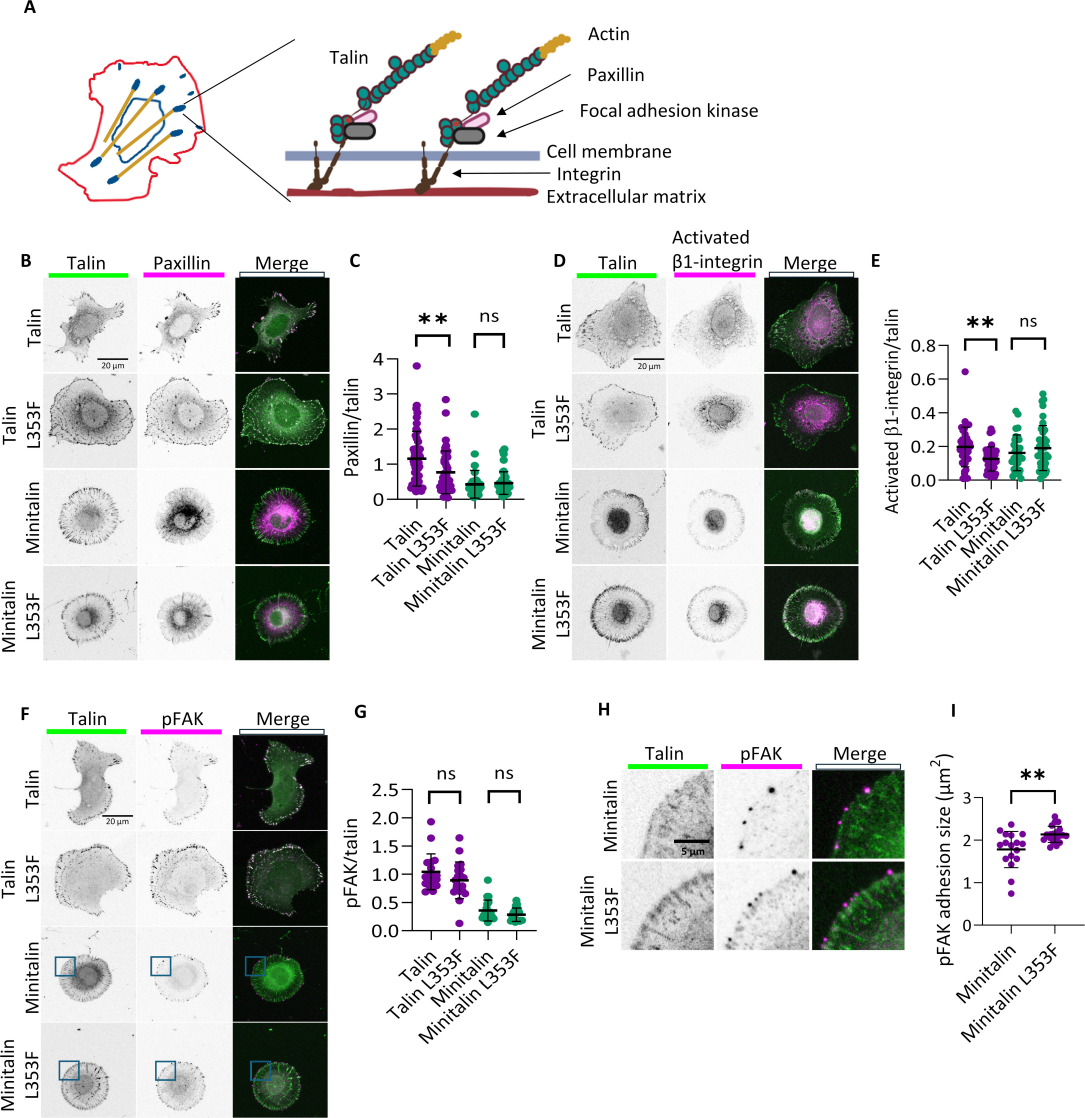
L353F mutation affects integrin adhesion complex composition. **(A**) Schematic structure of a focal adhesion indicating the components which were quantified to observe their amount and colocalization with talin-1. (**B**) Confocal images of paxillin quantification indicating the cellular expression of different talin-1 variants (left, green), endogenous paxillin (middle, magenta) and merged images depicting the paxillin and talin enriched adhesion regions (right, pink). (**C**) Paxillin/talin fluorescence intensity ratio values and measured differences between full-length and minitalin variants using *t*-test (**P<0.01). Each dot refers to an individual cell and the mean of the paxillin/talin ratio from 5 to 6 adhesion points from individual cells (*n* = 25–27 cells, 4–6 independent measurements). (**D**) Confocal images of activated integrin quantification. Cells expressing talin-1 variants (left, green), cells stained for activated β1 integrin (middle, magenta) and merged images showing analyzed adhesions (right, white). **E**) Activated β1 integrin/talin fluorescence intensity ratio values; each point represents the mean intensity from 5 to 6 adhesions from individual cells. The difference between full-length talin-1 and minitalin variants were measured using the *t*-test (**P<0.01) (*n* = 36–45 cells, 5–7 independent measurements). (**F**) Confocal images of pFAK quantification which includes cells expressing different variants of the talin-1 (left, green), followed by localized phosphorylated focal adhesion kinase (pFAK) (middle, magenta) and merged images showing the talin-1 and pFAK-rich focal adhesion regions (right, white). (**G**) Quantification of pFAK/talin fluorescence intensity ratio in focal adhesions. An individual dot represents the mean of the ratios from 5 to 6 adhesion points from each individual cell (*n* = 15–20 cells, 4–6 independent measurements). (**H**) Closer view of pFAK spots in minitalin-expressing talin-KO cells. Minitalin (left, green), pFAK (middle, magenta) and merged views (right, white). **I**) Quantification of the adhesion size from cells stained for phosphorylated focal adhesion kinase. Each dot represents an individual cell (total number of cells *n* = 19, 12–21 independent adhesions from individual cells).

**Figure 4:**
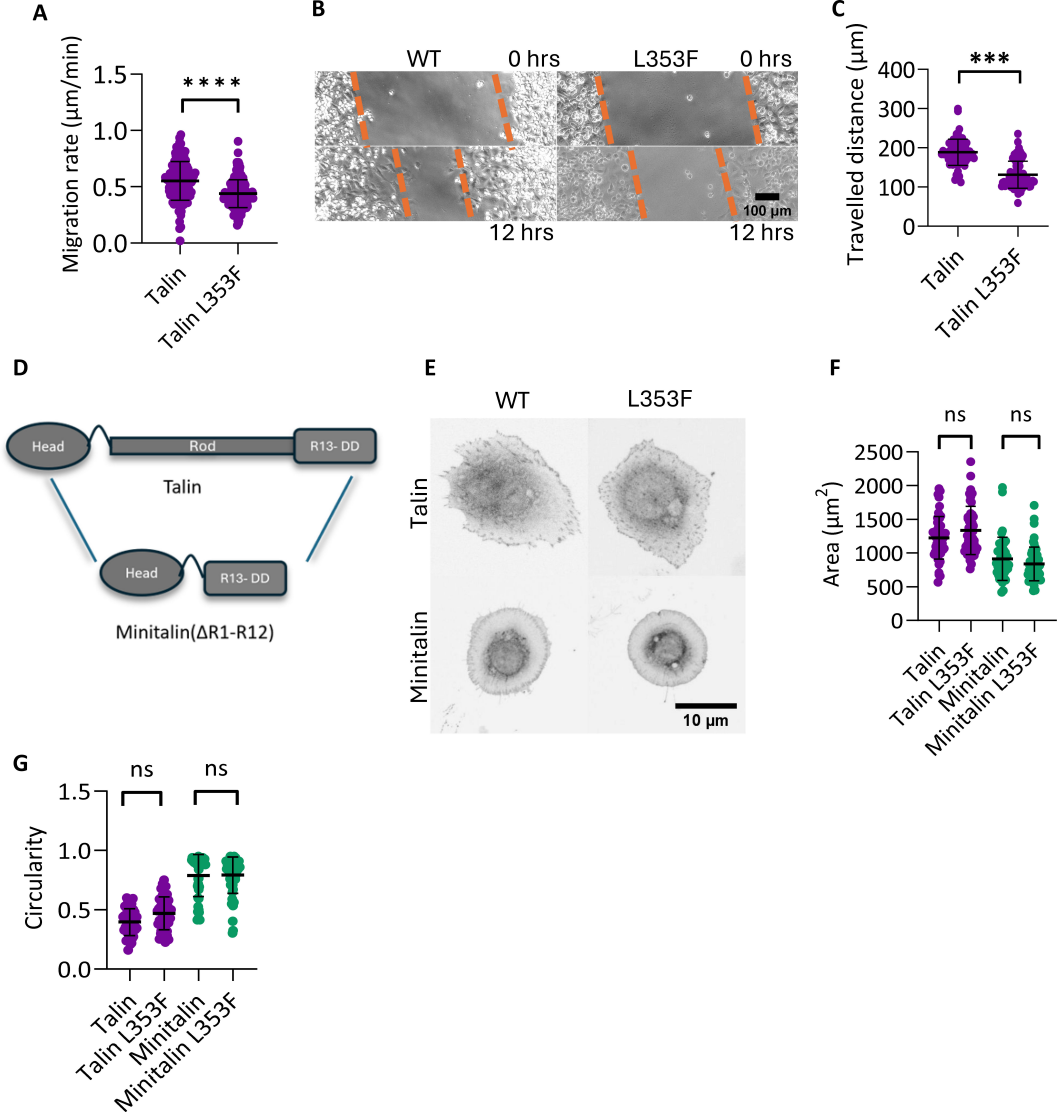
Talin-1 L353F mutation influences cell movement and wound healing but not cell morphology. **(A**) The migration rate measured in talin-KO cells expressing full-length WT and L353F talin-1, each dot corresponds to an individual cell. Total analyzed cells (n) = 100–150. Unpaired *t*-test, ****P<0.0001. (**B**) Brightfield images of wound healing assay for talin-KO cells expressing wildtype talin-1 and talin-1 L353F. The first row represents images from the initial time point (0 h), where the wound of 800 µm was made. Orange lines show the width of the wound. Images below show the migrated distance by cells in 12 h. (**C**) Quantification of B) showing the measured traveled distance after 12 h. Each point denotes an individual cell. Total analyzed cells (N) = 65–70. Unpaired *t*-test, ***P<0.001. (**D**) Schematic showing the domain structure of the talin-1 and minitalin constructs. (**E**) Confocal images of cells expressing Talin-1 WT, Talin-1 L353F, Minitalin WT and Minitalin L353F, indicating similar morphology between WT and mutant expressing cells. (**F**) Cell area measured for cells expressing each variant of talin-1. Each dot corresponds to an individual cell, total number of cells (n) = 30–35. (**G**) Circularity measured for cells expressing the different variants of talin-1. A circularity of 1 indicates a perfect circle, whereas 0 indicates an elongated shape. Unpaired *t*-test, non-significant *P* value denoted by ns.

Having shown a clear effect of the L353F mutation on the protein itself, we next wanted to characterize the influence of the mutation on cell movement and morphology in cultured cells. We designed plasmid constructs for recombinant expression of mutated and WT versions of full-length talin-1. Proteins were expressed in talin-knockout (Talin-KO) mouse kidney fibroblast cells [31]. In the absence of talin expression, these cells are unable to spread and polarize [31]. Transfection with both talin-1 expression plasmids restored an adherent phenotype, and protein expression levels were seen to be similar with each construct, and no signs of enhanced proteolytic activity were observed due to the L353F variant (Supplementary Figure S1). WT and mutant protein were both localized to focal adhesions (Figure 3B), and the observed cytoplasmic fraction of the L353F protein was slightly lower (Supplementary Figure S6). However, cells expressing talin-1 L353F were found to be migrating slower (average speed 0.42 µm/min with SD: 0.12) than the WT talin-1 expressing cells (0.54 µm/min with SD: 0.17) when random migration speed was measured (Figure 4A). Furthermore, in a wound healing assay, cells expressing talin-1 L353F were slower, traveling 138 µm on average in 12 hours as compared with WT cells that traveled 193 µm (Figure 4B C). Given the importance of actin in cell movement, we looked at the architecture of perinuclear actin fibers in the cell (Supplementary Figure S5A). No noticeable differences in the actin coherence were seen between WT and L353F expressing cells (Supplementary Figure S5B). To confirm that both talin forms support cell proliferation, we measured cell division events in live microscopy data and observed that cells expressing talin-1 L353F divided with equal pace compared to those expressing WT talin-1 (Supplementary Figure S7).

The L353F mutation is located in close proximity to the talin-1-integrin interface and therefore is likely to perturb talin-1 functions associated with the head domain. Talin-KO cells expressing talin-1 head alone are not able to form proper integrin adhesion complexes and spread minimally [32], limiting the possibilities to quantify the functional characteristics of talin-1 mutations. Therefore, to specifically focus on talin-1 head-related functions in a context where talin-1 can still make a mechanical linkage between the integrin and the cytoskeleton, we used our previously characterized ΔR1-R12 truncated ‘minitalin’ (Figure 4D). Without the talin-1 rod components allowing force-dependent growth and maturation of the adhesions, cells carrying minitalin cannot form completely matured focal adhesions and can be used as a tool to ‘freeze’ the adhesion complex in a non-matured state allowing examination of normally highly dynamic events in what amounts to a paused state. Therefore, minitalin lacking domains R1-R12 expressed in talin-knockout cells causes a spread but non-polarized phenotype that is unable to migrate [22,32]. Cell morphology was unchanged in the presence of L353F, both in cells expressing full-length and minitalin (Figure 4E–G).

### L353F mutation leads to changes in the adhesion composition

Integrin-mediated adhesion complexes are dynamic structures whose maturation from early focal complexes into mature focal adhesions is regulated by complex biochemical and mechanical signals. Mature focal adhesions consist of hundreds of proteins with integrins, talin-1, paxillin, and focal adhesion kinase (FAK) acting as key structural and signaling proteins (Figure 3A). To study how the L353F mutation affects adhesion maturation, we quantified the amount of these key proteins within mature FAs. We found that in talin-KO cells expressing talin-1 with the L353F mutation, there was less paxillin (Figure 3B C) and less active β1 integrin within the adhesions (Figure 3D E) compared with cells expressing WT talin-1. In contrast, the L353F mutation did not affect levels of activated FAK at adhesions as evaluated by autophosphorylation at tyrosine 397 (pFAK) (Figure 3F G). However, in cells expressing the minitalin versions, pFAK staining revealed nascent filopodia-like dots in the tips of the protrusions from the cell membrane (Figure 3H). Quantification of these pFAK positive dots showed that these structures are significantly larger (mean adhesion size = 2.1 µm^2^) in cells expressing the L353F mutation (Figure 3) as compared with cells expressing WT talin-1 (mean adhesion size = 1.7 µm^2^). These peripheral pFAK-rich clusters are associated with the truncated form of talin-1 (minitalin) and were not detected in cells expressing full-length talin-1 forms. The precise mechanism behind this finding remains unclear, but this suggests that the adhesion maturation process is altered due to the mutation.

## Discussion

Talin-1 is a central integrin adapter protein, and knockout studies in animal models have shown its critical role in animal development [33]. While widely studied *in vitro*, only limited information is available regarding the consequences of talin-1 mutations *in vivo* and in humans. Here, we report the identification of a patient with a novel talin-1 variant, L353F. We provide a complex medical case report and link these clinical symptoms to changes at the protein and cellular level. While modern computational methods facilitate structural analysis, predicting the phenotypical consequences at the levels of tissues and complete organisms remains a major challenge. Therefore, we have systematically evaluated the functional characteristics of this new talin-1 variant and how it is associated with clinical symptoms.

The L353F variant reported here is located in close proximity to the mutation analyzed in another case study we recently reported and causes a similar phenotype. The *de novo* talin-1 variant P229S we identified recently [22] was from a patient with a complex phenotype, including thrombocytopenia, leukopenia, congenital cataracts, eczema, and intermittent sinusitis, otitis media and bronchitis. The P229S mutation, also located in the talin-1 head, sits in the interface between the F2 and F3 domains and perturbs the crucial events of talin-1 head interacting with integrin and the plasma membrane, leading to defects in cell adhesion and migration. The detailed report of this new patient harboring the L353F variant helps to better understand the clinical spectrum of manifestations in patients with variants in talin-1 and may support identification of novel cases in the future.

Indeed, these two variants, P229S and L353F, share a lot in common: 1) both mutations are situated in the talin-1 head F2F3 domains; 2) cells bearing these mutations show defects in cell migration and integrin activation; 3) both patients are suffering from thrombocytopenia, leukopenia, congenital cataract, and recurring episodes of bronchitis. However, the P229S variant seems to manifest in additional complications, suggesting that its impact on talin-1 function is more severe. However, these results suggest the presence of a set of phenotypes caused by talin-1 mutations that affect the talin-integrin interaction. Table 1 summarizes the symptoms observed in these patients, highlighting similarities between the cases.

We observed talin-1 L353F expressing cells to have a lower amount of activated β1 integrins. Similarly, the amount of adhesion-localized paxillin decreased. These findings indicate that the mutation interrupts the tight regulation of the integrin activation process—the central role of talin in integrin activation has been observed in numerous previous biochemical and cell biology studies [29–31]. Our findings further support the link between defects in talin-1-mediated integrin activation and severe medical symptoms. These findings are in line with previous studies where integrin has been found to be a central player in processes such as leukocyte attachment, cell–matrix adhesion, and phagocytosis [34]. Moreover, we observed more extensive pFAK clusters in the filopodia-like structures in minitalin L353F expressing cells compared with cells expressing WT minitalin. A previous study by Jacquemet et al. suggests that FAK is normally present only in a subset of filopodia [35]. We speculate that due to the absence of WT talin-integrin association, Myosin X is dominating in filopodia tips and recruits activated FAK.

Talin-1 is essential for activation of platelet integrins β1 and β3 [36–39]. Fong et al. also observed that talin-1 is a target of calpain cleavage in platelets, and inhibition of calpain impaired clot contraction [36]. Knockout of the *TLN1* gene in platelet precursor megakaryocytes has been reported to cause spontaneous hemorrhage and pathological bleeding [37]. Petrich et al. also found that the absence of talin-1 in platelets leads to hindrance of platelet aggregation and adhesion [37]. Therefore, our observations associating the mutation L353F with defects in integrin activation and in recruitment of integrin adhesion complex components may help explain the potential contribution to the thrombocytopenia and other blood issues observed in the patient (Table 1).

Proteins involved in focal adhesions are known to link with disease conditions [40]. For example, Kindler syndrome, a form of skin disease, is caused by mutations in the kindlin gene (*FERMT1*) [41,42]. Kindlin is an adaptor protein that has been found to be responsible for the stability of the talin-1 and integrin bond [43]. We observed skin-related issues associated with L353F and talin P229S [22] linking talin-1 variants to skin diseases, which appears reasonable when considering the close collaboration between talin-1 and kindlin [31,44].

In summary, our findings identify a patient with a *TLN1* mutation which further supports the case for talin-1 as a disease-associated gene. We suggest that the L353F mutation within the talin-1 F3 domain is responsible for subtle disturbance of the talin-1 structure and function that leads to perturbed focal adhesion maturation and cell movement. The multifaceted symptoms observed in the patient carrying the mutation reflect the central role of talin-1 and the broad overlapping range of clinical symptoms seen with both the L353F and P229S variants demonstrates the necessity for normal talin-1 function in healthy physiology.

## Materials and methods

### Protein expression and purification

Mouse talin-1 F2F3 domains (196-400) WT and with the L353F mutation were synthesized as codon-optimized synthetic genes in pET151 (GeneArt, Life Technologies).

BL21(DE3) competent cells were transformed with the relevant plasmid and grown in lysogeny broth, supplemented with 100 µg/ml ampicillin at 37°C until the OD_600_ reached 0.6, whereupon expression was induced with 0.4 mM IPTG and the proteins expressed overnight at 20°C. Cells were harvested by centrifugation, resuspended in lysis buffer (50 mM Tris-HCl pH 8, 250 mM NaCl, 5% v/v glycerol) at 5 ml per gram of cells and stored at -80°C.

Proteins were purified by nickel-affinity chromatography, followed by cation exchange chromatography. Briefly, cells were thawed and supplemented with 1 mM PMSF and 0.2% v/v Triton X-100. Cells were lysed by sonication, and cell debris was removed by centrifugation. The supernatant was injected onto a 5 ml HisTrap FF column (Cytiva), after which the column was washed with buffer containing 50 mM Tris-HCl pH 8, 600 mM NaCl, 30 mM imidazole, 4 mM MgCl_2_, 4 mM ATP, 5% v/v glycerol, and 0.2% v/v Triton X-100. Bound protein was eluted across a 14 CV linear gradient of 0–300 mM imidazole, followed by 4 CV at 500 mM imidazole. Fractions containing F2F3 were pooled, diluted with 5 volumes of 20 mM sodium phosphate pH 6.5, and injected onto a 5 ml HiTrap SP cation exchange column (Cytiva). Bound proteins were eluted across a 14 CV linear gradient of 0–600 mM NaCl, followed by 4 CV at 1 M NaCl. Fractions containing F2F3 were dialyzed against PBS pH 7.4, snap-frozen in LN2, and stored at −80°C.

### Nuclear magnetic resonance spectrometry (NMR)

Cells were grown in ^15^N-labeled 2M9 minimal media (40 mM Na_2_HPO_4_, 20 mM KH_2_PO_4_, 10 mM NaCl, 100 µM CaCl_2_, 1 mM MgSO_4_, 10 ml BME vitamin solution (Sigma-Aldrich), 0.4% (w/v) glucose, 0.1% (w/v) ^15^N-labeled NH_4_Cl) and supplemented with 100 µg/ml ampicillin. ^15^N-labeled proteins were expressed and purified as above and prepared at 150 µM final concentration in 20 mM phosphate buffer pH 6.5, 50 mM NaCl, 2 mM DTT, 5% (v/v) D_2_O. ^1^H,^15^N TROSY spectra were collected at 298 K on a Bruker Avance III 700 MHz NMR spectrometer equipped with cryoprobe. All data were processed using TopSpin (Bruker) and analyzed with CCPN Analysis [45]. Peaks in the WT spectrum were identified by comparing them with the assigned F2F3 spectrum [26,46].

### Circular dichroism (CD)

CD was performed using a JASCO J-1100 spectropolarimeter with 20 µM protein samples in PBS pH 7.4 supplemented with 0.5 mM TCEP. The far-UV spectra were an average of six scans collected between 198 and 300 nm, at 50 nm/min, 1 nm step resolution and a bandwidth of 1 nm. Thermal denaturation data were collected by increasing the temperature from 20 to 90°C at a rate of 1 °C/min. The CD signal at 222 nm was measured at 0.5°C intervals using a 1 nm bandwidth. Melting temperatures were calculated by fitting a Boltzmann distribution to the data in OriginPro (OriginLab Corporation).

### Size exclusion chromatography with multi-angle light scattering (SEC-MALS)

Protein samples were prepared at 4 mg/ml in SEC buffer (PBS pH 7.4, 0.5 mM TCEP). 100 µl of either WT or L353F talin-1 F2F3 was injected onto an S200 10/300 increase (Cytiva) size exclusion column (equilibrated in SEC buffer) at 0.75 ml/min using an AKTA Pure (Cytiva). Inline 8-angle light scattering and refractive index data were collected using a Heleos II DAWN 8 + and an Optilab tREX, respectively (Wyatt Technology). Data were analyzed using ASTRA (v6.1.2.84; Wyatt Technology) and plotted using OriginPro (OriginLab Corporation).

### Dynamic/static light scattering (DLS/SLS)

Protein samples were prepared at 8 mg/ml in PBS pH 7.4, 0.5 mM TCEP. 1.5-fold serial dilutions of WT and L353F talin-1 F2F3 were prepared and loaded into nanoDSF-grade standard capillaries (PR-C002; NanoTemper). DLS/SLS data were collected using a Prometheus Panta (NanoTemper), performing 10 high sensitivity scans of each capillary at 25°C. The 2^nd^ virial coefficient (B_22_) was determined using the instrument’s software (Panta Control v1.9; NanoTemper) and replotted in OriginPro (OriginLab Corporation).

### Fluorescence polarization (FP) assay

The β1A integrin (UniProt: P05556; residues: 752–798) cytoplasmic tail peptide containing a non-native N-terminal cysteine (C-KLLMIIHDRREFAKFEKEKMNAKWDTGENPIYKSAVTTVVNPKYEGK) was synthesized by GLBiochem (Shanghai). Maleimide-fluorescein dye (Thermo Fisher Scientific) was coupled to the peptide following the manufacturer’s protocol. Assays were performed in PBS pH 7.4, 0.01% v/v Tween-20, 0.5 mM TCEP in triplicate with a 2-fold serial dilution of protein and peptide at 500 nM. FP was measured using a Hidex Sense plate reader (Hidex) at 25°C (excitation: 485 ± 10 nm; emission: 535 ± 20 nm). Data were analyzed using OriginPro (OriginLab Corporation) and *K_d_* values were generated using a single site with non-specific binding equation.

### MD simulations

The PDB structure, 6VGU [27] was used as the starting conformation for MD: for both free F2F3 (residues 196–405) and complexed with integrin β3 tail (residues 740–750 for integrin).

Alchemical free energy calculations were prepared using PMX [47] and performed with Gromacs [48] at Mahti supercomputer, CSC, Finland. The Amber99SB*–ILDN force field [49] and TIP3P water model in 0.15 M NaCl solution were used. Each system was energy minimized for 10,000 steps and then equilibrated for 1 ns using harmonic position restraints on all heavy atoms of the protein. The temperature and pressure of the system were maintained at 298 K and 1 bar using the Berendsen algorithm [50] for the system equilibration, while V-rescale [51] and Parrinello–Rahman [52] algorithms were used for equilibrium MD and non-equilibrium morphing simulations. An integration time step of 2 fs was used in all the simulations. Each state of the system was run for 200 ns equilibrium MD for the folded state, while 100 ns for tripeptide resembling unfolded state of the mutant. 186 and 99 non-equilibrium morphing simulations were prepared for each physical state of the system for the folded and unfolded states, respectively, using snapshots captured from the equilibrium trajectories, linearly spaced from 15 to 200 ns for folded states and from 2 to 100 ns for unfolded states. Fast nonequilibrium simulations were morphing the system from one state to another in 200 ps. A soft-core potential [53] was used. The whole calculation, including system preparation, was repeated three times and the average free energy value was obtained.

### Cells, plasmid constructs, and cloning

In all experiments, talin-1 and talin-2 knockout cells (TLN1^−/−^ TLN2^−/−^ mouse kidney fibroblast; MEF-DKO cells) were used as described previously [28]. Cells were maintained at 37°C, 85–95% humidity, and 5% CO_2_. Dulbecco’s modified Eagle medium (DMEM) containing high glucose, GlutaMAX (ThermoFisher Scientific, Catalog number: 61965059) supplemented with 10% fetal bovine serum (FBS) (ThermoFisher Scientific, Catalog number: 10,270,106) was used in all experiments.

Expression plasmid constructs were designed using the SnapGene tool. Plasmids were cloned and produced through GenScript plasmid cloning service. Sequences of talin-1 were cloned into a modified pEGFP vector backbone (Clontech). Expression constructs with the C-terminal EGFP-tag used in this work are as follows: full-length WT talin-1 (1–2541 aa); mini-talin-1 (1–490 aa+2296–2541 aa; head-R13-DD domains); and the L353F variant of each construct.

### Transfection

Talin-KO cells were dissociated using TrypLE (Thermo Fisher Scientific, Catalogue number: 12604013) and centrifuged for 5 min at 150 ***g***. The cell pellet was resuspended in transfection R buffer and cells transfected with the following plasmid amounts; EGFP-talin-1 10 μg, EGFP-talin-1 L353F 10 μg, EGFP-minitalin 6 μg and EGFP-minitalin L353F 6 μg. In each transfection, 1.2 × 10^6^ cells were used. A Neon electroporation system (Product No: 10431915, Fisher Scientific) with the parameters 1350 V, pulse width 40, and single pulse was used for the transfections.

### Total migration assay and migration rate analysis

To analyze the total cell migration, transfected cells were plated on substrate [10 μg/ml human plasma fibronectin (Produced in Tampere University)] coated 12-well plates, DMEM + 10% FBS medium was added and cells kept for incubation overnight at 37°C, 85–95% humidity, and 5% CO_2_. After overnight incubation, DMEM media was removed from the wells and new media added. To image total cell migration, an EVOS FL auto microscope (Thermo Fisher Scientific) was used. Time-lapse images with an interval of 30 min for 24 h were captured. Traveled distance by cells was quantified using the MTrackJ plugin in ImageJ (Fiji) [54,55]. Individual cells from the single focal plane were selected using the add option and traveled distance tracked in each time frame. Values were recorded in Excel and migration speed was calculated [travelled distance/time (μm/min)]. Calculated values were plotted using GraphPad Prism 10.3.1.

### Wound closure assay

For the quantification of wound closure, cells were transfected with the full-length EGFP-talin-1 constructs. Transfected cells were plated on 10 μg/ml fibronectin and incubated to reach high confluency (90%) in a 24-well plate for 48 h in the cell culture incubator. The artificial wound (approximately 800 μm) was created using a 100 μl pipette tip. After making the wound, the wells were washed with PBS to remove dead and detached cells. New DMEM + 10% FBS medium was added to the wells. Wound closure was imaged using an EVOS FL auto microscope for 12 h. Images of cells from a single plane were taken using a 10× objective at 20 min intervals. Acquired images were analyzed manually using ImageJ (Fiji) by using a freehand line tool measuring the distance that cells traveled in 12 h from the starting point.

### Immunofluorescence

Immunofluorescence was used for the quantification of the amount of paxillin, phosphorylated focal adhesion kinase, and activated β1 integrin. Each protein was individually labeled in separate experiments. Talin-KO cells were maintained in similar conditions and transfected using EGFP-talin-1 constructs. Transfected cells were added to coverslips coated with fibronectin (10 µg/ml) substrate and incubated overnight at 37°C, humidity 85–95%, and 5% CO_2_ in 24 well plates. After incubation, media from the samples was removed and the cells were washed with PBS. Cells were then fixed using 1 ml of 4% paraformaldehyde on coverslips. Fixed cells were incubated in permeabilization buffer (0.2% triton X-100 in PBS) for 10 min. The permeabilization buffer was removed and cells were washed with PBS for 1 min. Cells were then incubated in primary antibody solution diluted in 3% BSA in PBS (Supplementary Table S1) against each protein for 45 min. The primary antibody solution was removed, and cells were washed in PBS for 1 min. Cells were further incubated in secondary antibody solution [Secondary antibodies conjugated with fluorophores (Supplementary Table S1) diluted in 3% BSA in PBS] for 45 min. The secondary antibody solution was removed, and cells were washed using PBS for 1 min. Cells were then quickly washed using milliQ H_2_O, and coverslips were mounted on imaging slides using mountant (ProLong™ Diamond Antifade Mountant, Catalog number: P36961).

### Fluorescence imaging and image analysis

Immunolabeled samples were imaged with a Zeiss LSM800 confocal microscope using a plan-apochromat (63 x/1.4 NA) oil objective lens.

To quantify the amount of paxillin in the integrin adhesion complex, cells expressing different EGFP-talin-1 versions were labeled for paxillin and a secondary antibody conjugated with Alexa Fluor 568 was used. Alexa Fluor 568 was excited using a 561 nm laser with 1% intensity. Fluorescence was detected using 50 µm pinhole size and 660 V detector gain. EGFP talin-1 was located using a 488 nm laser (1% intensity, 50 µm pinhole size and 650 V detector gain). To quantify the amount of phosphorylated FAK, cells were stained using an anti-pFAK antibody and subsequently stained with secondary antibody conjugated with Alexa Fluor 568 using similar parameters to excite the Alexa Fluor 568 and EGFP talins. For the quantification of activated β1 integrin cells, the cells were stained using anti-β1 integrin antibody, subsequently stained with secondary antibody conjugated with Alexa Fluor 568 and excited using 1% intensity. Fluorescence was detected using a 50 µm pinhole size and 700 V detector gain. EGFP talin-1 in these cells was excited using a 488 nm laser with 1% intensity and fluorescence was detected using 50 µm pinhole size and 700 V detector gain.

Images were collected at 768 × 768-pixel size with a bit depth of 8. Collected images were quantified using ImageJ (version 1.52 n). To quantify the ratios of paxillin, pFAK, and activated β1 integrin to talin-1, images were split into separate channels then converted to the sum intensity projection (Image > stacks default Z project > Sum slices). To select the adhesions, the elliptical brush tool was used (size 8 pixels). Each spot was selected from the individual focal adhesions and location was added to the ROI manager tool. Fluorescence intensity values were quantified using the measure option. These values were plotted using GraphPad Prism 10.3.1.

### Cell morphology analysis

Cell morphology was analyzed using images acquired for the focal adhesion protein amount quantification. Single cells from the images were measured using freehand selection in ImageJ (ImageJ > Analyze default set measurements > area and shape descriptors). Manual lines were drawn around the cell membrane and the cell area, and cell shape descriptors were measured using ImageJ > Analyze default measure. Measured values were collected in Excel and plotted in GraphPad Prism 10.3.1.

### Adhesion size analysis

The size of nascent adhesions, present in cells stained for pFAK, was analyzed using ImageJ. Confocal images of cells expressing minitalin and minitalin L353F collected to analyze the pFAK amount were used for the analysis. The image stacks were split into individual slices. Brightness and contrast controls were used to set an intensity threshold of 100, and adhesions with values below this were eliminated. Images were then converted to binary (toolbar > process default binary > make binary). Nascent adhesions were selected using the freehand selection tool. Components around the selections were cleared using (Edit > Clear Outside). The adhesion dots were then enlarged to create a mask (Process > Binary default dilate and Edit >select default create mask). The mask was then copied to the original image, and the ROI was added to the ROI manager (Analyze > tools default ROI Manager). The area of individual adhesions was measured using (Analyze > tools default ROI manager > Split default Multi Measure). The measured values were plotted using GraphPad Prism 10.3.1.

### Actin orientation analysis

To analyze the actin coherency coefficient, Talin-KO cells were transfected as previously described. Transfected cells were added to coverslips coated with fibronectin (10 µg/ml) substrate and incubated overnight at 37°C, humidity 85–95%, and CO_2_ 5% in 24 well plates. The cells were washed and permeabilized as previously described. Samples were incubated in Phalloidin-Alexa Fluor 647 solution (phalloidin diluted in 3% BSA and PBS) for 30 min (Supplementary Table S2). Samples were washed with PBS, rinsed in milliQ H_2_O, and mounted on microscopy slides using mountant (ProLong™ Diamond Antifade Mountant, Catalog number: P36961).

Prepared samples were imaged with a Zeiss LSM800 confocal microscope using a plan-apochromat (63 x/1.4 NA) oil objective lens. Cells were detected using a 488 nm laser (1% intensity, 47 μm pinhole size, and 650 V detector gain) for the GFP-labeled talins and a 640 nm laser (0.7% intensity, 56 μm pinhole size, and 620 V detector gain) for the actin-Alexa fluor 647. Each cell was scanned using a Z stack. Images were collected in 768 × 768-pixel size with a bit depth of 8.

The OrientationJ plugin in ImageJ was used for the measurement of actin orientation in collected images. The EGFP talin-1 and Alexa Fluor 647-actin channels were separated. The actin stack was split into slices (Image > stacks default stacks to images) and a single slice with higher actin fluorescence intensity was used. In the selected slice, a mask was drawn around the actin fibers present over the nucleus, and coherency was measured (Plugins > OrientationJ - > orientationJ measure > measure). Coherency values were plotted using GraphPad Prism 10.3.1.

### SDS-PAGE and Western blotting

To analyze changes in talin-1 expression due to mutation, SDS-PAGE and Western blot was performed. MEF-DKO cells were first transfected using the previously described parameters. Transfected cells were plated on 10 µg/ml fibronectin substrate 12-well plates for 24 h at 37°C. Cells were lysed in 60 µl 2 x SDS (sodium dodecyl sulfate in MilliQ water). Lysates were boiled for 15 min at 100°C. 10 µl of each sample was loaded onto precast polyacrylamide gels (BIO-RAD, Any kD™ Mini-PROTEAN® TGX™ Precast Protein Gels, 15-well, 15 µl #4569036). Gels were run for 60 min in SDS running buffer (25 mM tris, 190 mM glycine, 0.1% SDS) at 170 V. Proteins were transferred onto nitrocellulose membranes (BIO RAD, Transblot turbo, Mini 0.2 µm Nitrocellulose) using Transblot turbo system (BIO-RAD) for 10 min at 25 V.

The blot was blocked with BSA (5% BSA+TBS) for 30 min at room temperature. Anti-GFP primary antibody (Supplementary Table S3) was added to the BSA, and the blot was incubated overnight at 4°C and washed for 3 × 10 min using 0.05% v/v tween 20 in TBS. The secondary antibody, donkey anti-goat IRDye 680RD (LI-COR biosciences) (Supplementary Table S3), with 5% BSA+TBS was added to the blot and incubated for 1 h at room temperature. The blot was washed using 0.05% Tween 20 + TBS for 3 × 10 min and imaged with the LI-CORE Odyssey CLx system (LI-COR biosciences).

## Supplementary material

Supplementary data

## Data Availability

The original data is available from corresponding authors upon request. The plasmids used for protein expression in mammalian cells will be available through the Addgene (https://www.addgene.org/Vesa_Hytonen/) (56). The expression plasmids for bacterial expression of F2F3 and F2F3(L353F) will be available in Addgene (https://www.addgene.org/Ben_Goult/) (57).

## Abbreviations

B22: second virial coefficient
BSA: bovine serum albumin
ECM: extracellular matrix
ES: exome sequencing
F2: subdomain within the talin-1 head
F3: subdomain within the talin-1 head
FAK: focal adhesion kinase
FAs: focal adhesions
FP: fluorescence polarization
K_d_: dissociation constant
MD: molecular dynamics
MEF-DKO: mouse embryonic fibroblast - double (talin) knockout
OFC: occipitofrontal circumference
PBS: phosphate-buffered saline
PolyPhen-2: polymorphism phenotyping v2
REVEL: Rare Exome Variant Ensemble Learner
ROI: region of interest
R1–R13 13: α-helical rod domains of talin-1
SD: standard deviation
SDS: sodium dodecyl sulfate
SEC-MALS: size exclusion chromatography with multi-angle light scattering
SLS: static light scattering
TLN1: talin-1 (gene)
Talin-KO: talin-knockout
WT: wildtype
gnomAD: Genome Aggregation Database
pFAK: phosphorylated focal adhesion kinase
