# Supplementary material for: De novo talin-1 variant L353F connects multifaceted clinical symptoms to alterations in talin-1 function
Source: Biochem J. 2025 Sep 17;482(18):1355–70. doi: 10.1042/BCJ20253128 (PMC12599238; doi:10.1042/BCJ20253128)
Supplement: Supplementary data [file bcj-482-18-BCJ20253128-s001.docx]

**Supplementary Information**

**Supplementary Figure 1:**

**
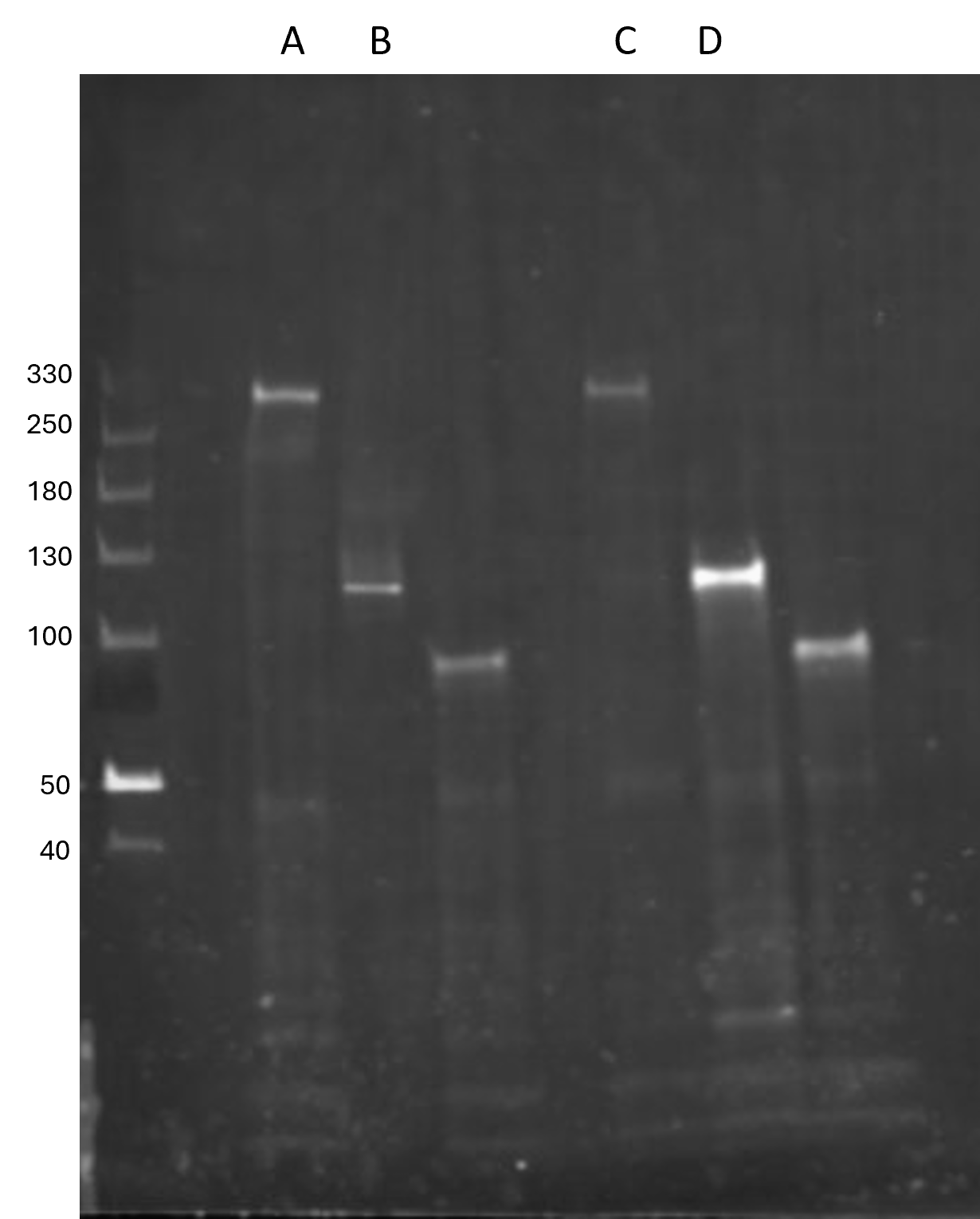
**

**Supplementary Figure 1: Expression of talin-1 is not influenced majorly by the L353F mutation.** Western blot showing the protein expression in MEF-DKO cells. **A)** Talin-1 **B)** Minitalin **C)** Talin-1 L353F **D)** Minitalin L353F.

**Supplementary Figure 2:**

**
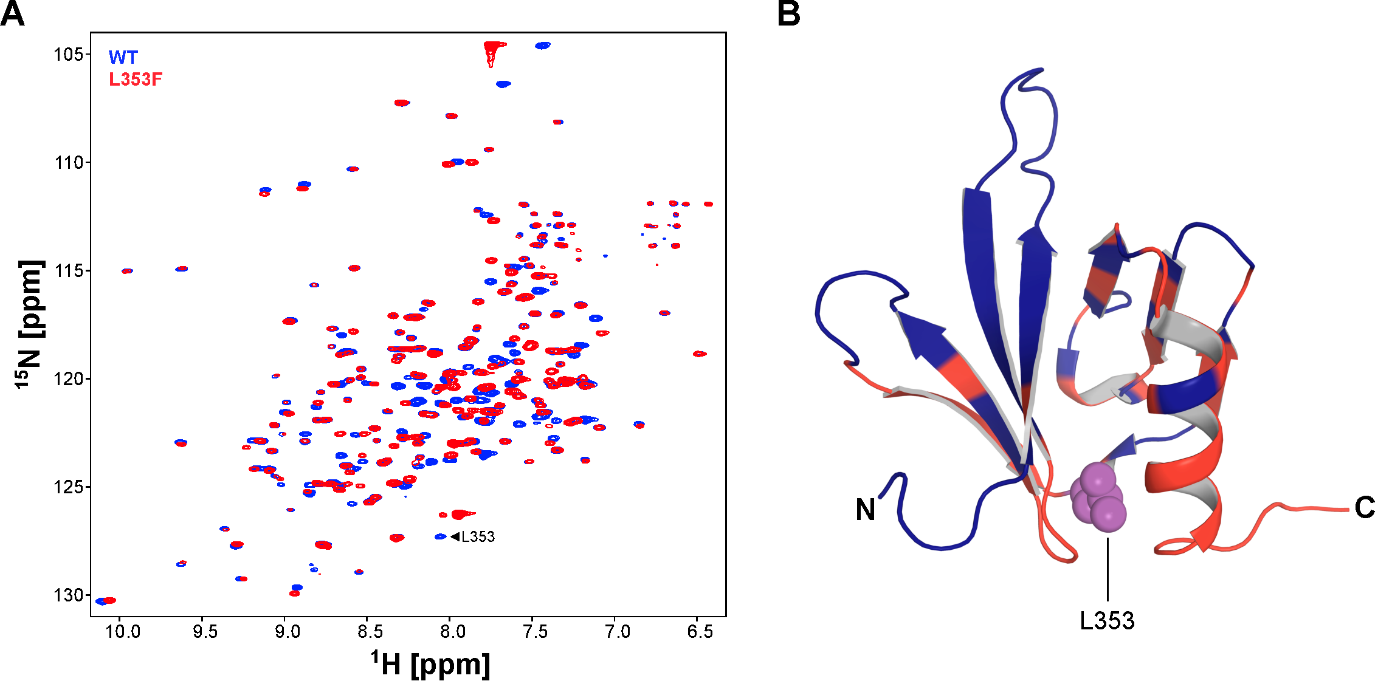
**

**Supplementary Figure 2: NMR analysis of WT and L353F talin-1(F2F3). (A)** ^1^H,^15^N TROSY spectrum of F2F3 WT (blue) and L353F (red). The L353 peak is indicated in the WT spectrum. (B) Peak shifts upon introduction of the L353F mutation mapped onto the F3 domain of the crystal structure of F2F3 (PDB: 1MIX; 29). Peaks that have shifted in the mutant spectra are colored red. L353 is shown in magenta.

**Supplementary Figure 3:**

**Supplementary Figure 3: Expanded view of SEC-MALS data (figure 2D).** Light scattering data (solid lines) and calculated masses (points) for WT (blue) and L353F (red) at 4 mg/mL.

**Supplementary Figure 4:**


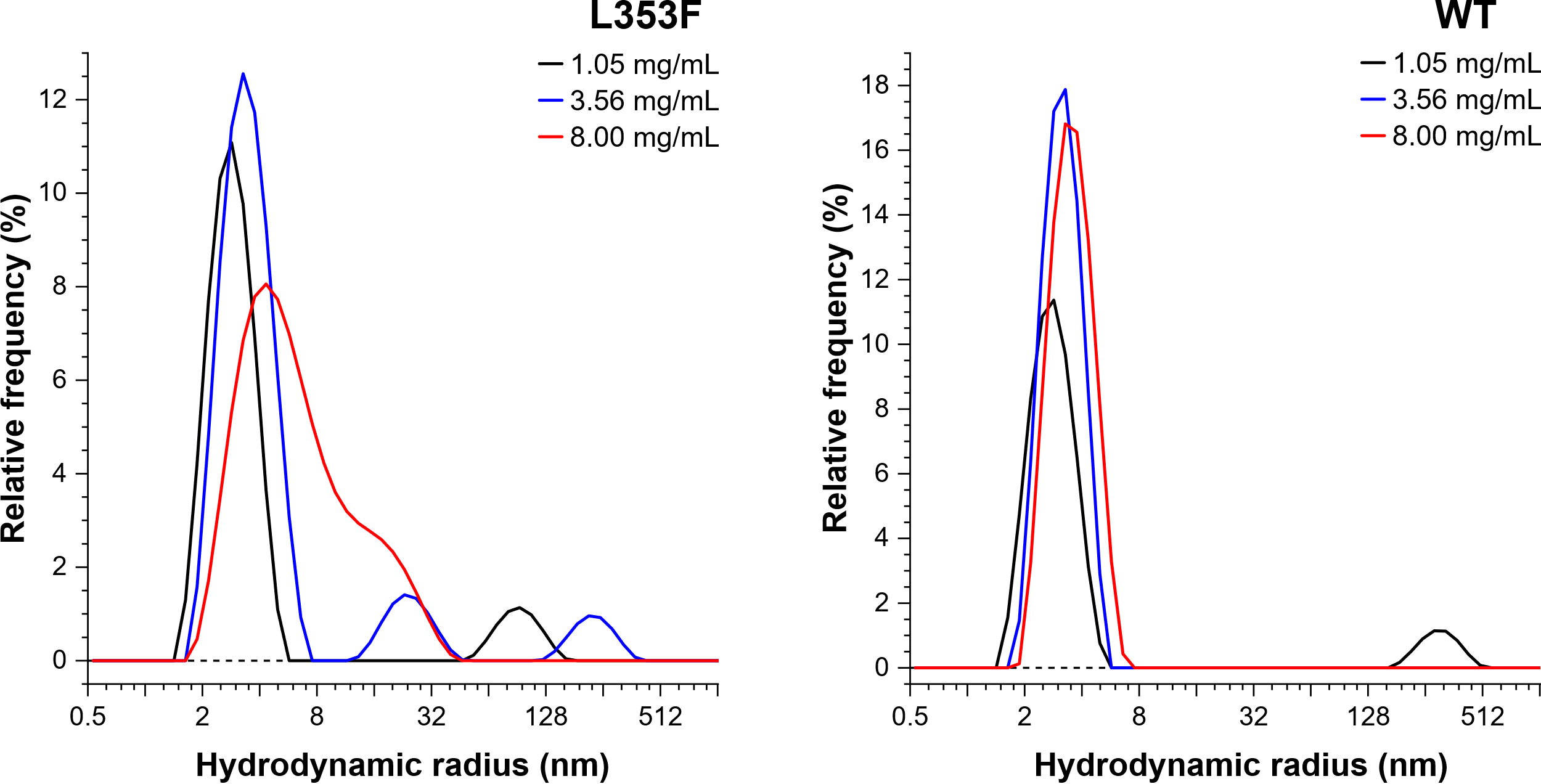


**Supplementary Figure 4: DLS size distribution of samples used for B22 determination.** Size analysis of talin-1 F2F3(L353F) (A) and WT (B) at 1.05 mg/mL (black), 3.56 mg/mL (blue) and 8 mg/mL (red) were performed on a Prometheus Panta (NanoTemper) prior to B22 analysis (Figure 2E). Data was analyzed in the instrument’s analysis software (Panta Analysis, v1.9) and the intensity distributions were plotted in OriginPro (OriginLab).

**Supplementary Figure 5:**

**
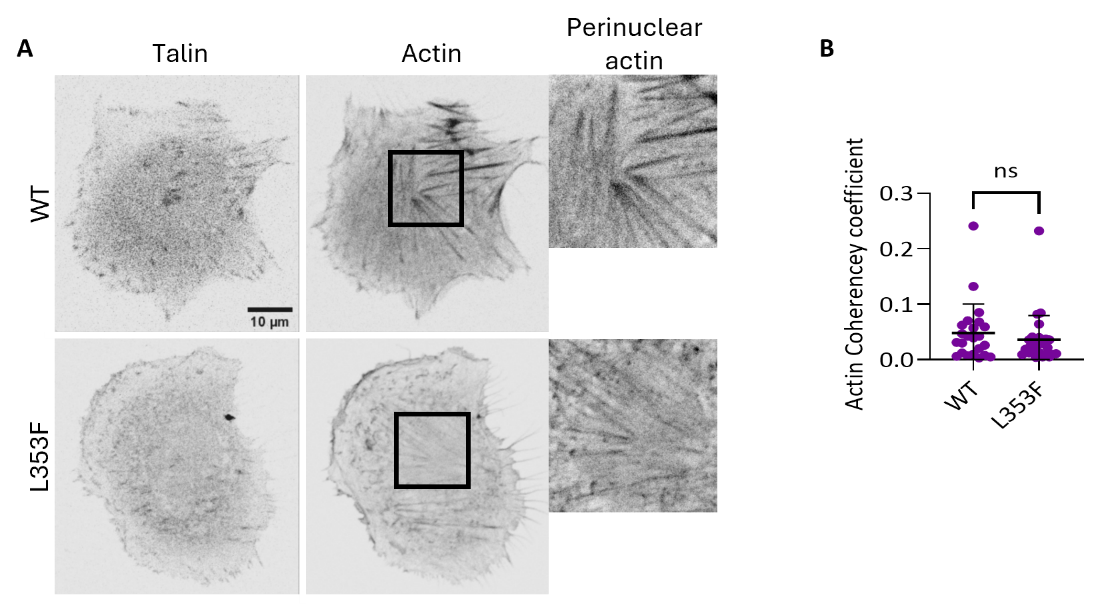
**

**Supplementary Figure 5: Talin-1 L353F mutant does not affect the perinuclear actin coherence.** A) Confocal images of cells expressing WT (top) and L353F (bottom). The full length talin-1 (left) and the distribution of actin in the same cells (right) are shown. The box shows the analyzed regions over the nucleus. B) Coherency coefficient values of the actin filaments. Coherency designates the alignment of the nuclear actin from 0 to 1 (0 = random distribution and 1 = perfectly aligned filaments). Dots on the graph denote the individual cell (total analyzed cells (n) = 25 to 30). Differences between coherency values were measured using a t-test (ns = no significant difference).

**Supplementary Figure 6:**

**Supplementary Figure 6: Cytoplasmic talin-1 quantification. Cytoplasmic fluorescence signal was quantified from areas outside focal adhesions.** Total number of cells: 21 per construct. Each dot represents Talin1-EGFP fluorescence intensity from individual cell, difference measured using t-test (* = p value < 0.05).

**Supplementary Figure 7:**

**Supplementary Figure 7**: **Rate of cell proliferation is not influenced by L353F mutation.** The percentage of cells dividing during 24 hours live cell imaging is measured in talin-KO cells transiently expressing EFGP-fused talin-1 or talin-1 L353F. Total number of cells: 61 cells expressing talin-1 and 82 cells expressing talin-1 L353F. Difference is measured using t-test (ns = no significant difference).

**Supplementary Table 1:**

| **Antibodies** | **Dilution used** |  |
| --- | --- | --- |
| **Phosphorylated focal adhesion kinase (pFAK) antibodies:**  Primary antibody:  Anti-FAK (phospho Y397) rabbit antibody (Abcam, catalogue number: EP2160Y)  Secondary antibody:  Goat anti rabbit Alexa fluor 568 (Thermo scientific, catalogue number: A11011) | 1: 200  1:250 |  |
| **Paxillin immunofluorescence antibodies:**  Primary antibody:  Anti-Paxillin mouse polyclonal antibody (BD Biosciences, catalogue number: AB_397464)  Secondary antibody:  Goat anti mouse Alexa fluor 568 (Thermo Scientific, catalogue number: A32723) | 1:100  1:250 |  |
|  |  |  |
| **Integrin antibodies:**  Primary antibody:  Anti-Activated β1 integrin mouse antibody (CD29 clone) (BD Biosciences, catalogue number: 553715)  Secondary antibody:  Goat anti mouse Alexa fluor 568 (Invitrogen, catalogue number: A11004) | 1:100  1: 250 |  |
|  |  |  |

**Supplementary Table 2:**

| **Actin stain** | **Dilution used** |
| --- | --- |
| Phalloidin Alexa fluor 647  (Abcam, catalogue number: AB0020-500 ab176759) | 1:40 |

**Supplementary Table 3:**

| **Antibodies** | **Dilution used** |
| --- | --- |
| **Primary antibody**  Goat anti-GFP  (Sicgene, catalogue number: AB0020-500)  **Secondary antibody**  Donkey anti goat IRDye 680RD  (Li-Cor, catalogue number: 926-68074) | 1:1000  1:10000 |
